# Locus of (IL-9) control: *IL9* epigenetic regulation in cellular function and human disease

**DOI:** 10.1038/s12276-024-01241-y

**Published:** 2024-06-03

**Authors:** Aran Son, Ishita Baral, Guido H. Falduto, Daniella M. Schwartz

**Affiliations:** 1 Neuroscience Department, International School for Advanced Studies (SISSA), via Bonomea 265, Trieste, 34136 Italy; 2 Division of Rheumatology and Clinical Immunology, University of Pittsburgh, Pittsburgh, PA USA

**Keywords:** Epigenetics in immune cells, Interleukins

## Abstract

Interleukin-9 (IL-9) is a multifunctional cytokine with roles in a broad cross-section of human diseases. Like many cytokines, IL-9 is transcriptionally regulated by a group of noncoding regulatory elements (REs) surrounding the *IL9* gene. These REs modulate IL-9 transcription by forming 3D loops that recruit transcriptional machinery. IL-9-promoting transcription factors (TFs) can bind REs to increase locus accessibility and permit chromatin looping, or they can be recruited to already accessible chromatin to promote transcription. Ample mechanistic and genome-wide association studies implicate this interplay between IL-9-modulating TFs and *IL9 cis*-REs in human physiology, homeostasis, and disease.

## Epigenetic regulation orchestrates IL-9 transcriptional control

Interleukin 9 (IL-9), a protein that helps the body’s immune system, has been researched for its role in various diseases. However, the mechanisms regulating IL-9 is production are not completely known. A team of researchers, led by A. Son and D.M. Schwartz, have reviewed how IL-9 production is controlled, focusing on specific elements within the *IL9* gene and surrounding non-coding regulatory DNA. They reviewed existing studies, mainly in mice, and found several factors that bind to these elements and affect IL-9 production. They also discussed how *IL9* epigenetics are connected to human diseases, finding links with allergies, autoimmune diseases, and cancer. These discoveries could aid in creating new treatments for these diseases. The researchers concluded that more studies are needed to fully understand how epigenetic regulation of IL-9 modulates disease. This could lead to new treatment strategies for various conditions.

This summary was initially drafted using artificial intelligence, then revised and fact-checked by the author.

## Introduction

Interleukin 9 (IL-9), first discovered in the 1980s, is a pleiotropic cytokine with roles in type 2 immunity, autoimmunity, antitumor immunity, and other cellular processes^1^. IL-9 has diverse sources that include CD4^+^ T cells, CD8^+^ T cells, innate lymphoid cells (ILCs), mast cells, and basophils^1^. While the sources and functions of IL-9 are tissue- and disease-specific, T helper 9 (Th9) cells are the best characterized and most abundant source of IL-9, particularly in humans^1^. Indeed, IL-9 is the hallmark effector cytokine of Th9 cells and defines them as a discrete T helper subset^1^.

Like many other cytokines, IL-9 is controlled by noncoding regulatory elements (REs) surrounding the *IL9* gene^2–5^. These include the *IL9* promoter and several enhancer elements. Enhancers modulate transcription by forming 3D interactions, or loops, that recruit transcriptional machinery^5^. Some transcription factors (TFs) can act as “pioneer” factors, binding to a promoter or enhancer to increase chromatin accessibility within a locus, permitting the formation of loops and recruitment of downstream TFs^6^. This interplay between accessibility, chromatin remodeling, looping, and TF recruitment regulates IL-9 production through the actions of several REs within the extended locus^3,4,7,8^.

Because IL-9 regulation is best characterized in CD4^+^ T cells, most epigenetic IL-9 studies have focused on Th9 cells. Relative to other T helper subsets, Th9 cells display several unique features associated with *IL9* epigenetic regulatory mechanisms. Most strikingly, Th9 cells exhibit transcriptional and epigenetic instability, which may be a negative checkpoint on sustained STAT5- and STAT6-dependent bystander activation^2,9^. Stable circulating Th9 cells are not detected in healthy subjects but can be found in patients with inflammatory diseases, suggesting that *IL9* epigenetic regulation is aberrant in these individuals. This aberrant regulation could be related to cell-intrinsic differences or downstream of microenvironmental cues specific to the inflammatory microenvironment that alter *Il9/IL9* epigenetics in CD4^+^ T cells.

Here, we review fundamental mechanisms of epigenetic IL-9 regulation, which are largely derived from studies in murine models. We then analyze genome-wide association studies to extrapolate known links between IL-9 epigenetics and human disease. Finally, we review mechanistic evidence for IL-9 epigenetic regulatory mechanisms in disease pathogenesis, while describing ongoing and future areas of investigation in this field.

## Structure of the extended IL9 locus and the function of key regulatory elements

The *Il9/IL9* locus occupies an ~45-kb region comprising a promoter and several critical downstream and upstream *cis*-regulatory elements (REs) with enhancer activity^3,4,7,8,10^. One unique feature of the extended *Il9/IL9* locus is its dynamic accessibility in Th9 cells, which is associated with Th9 lineage instability^2^. Like other cytokine loci, the *Il9/IL9* locus becomes accessible during differentiation, with accessibility and TF binding beginning to increase within 12 hours of activation^6^. During differentiation, this programming of the *Il9/IL9* locus depends on a complex network of TFs that includes several TCR-induced factors. Once the extended locus has become accessible, a smaller set of TFs is critical for acute activation of the locus. We and others have shown that STAT5 and STAT6 have a critical role downstream of IL-2 and IL-4^2,11^. However, after removing TCR stimulation, accessibility and histone architecture change slowly over time, rendering the locus refractory to transcriptional activation. While the human *IL9* locus is more stable than the murine *Il9* locus, it also exhibits dynamic accessibility and TF binding^2^.

### The murine *Il9* locus

**Fig. 1:**
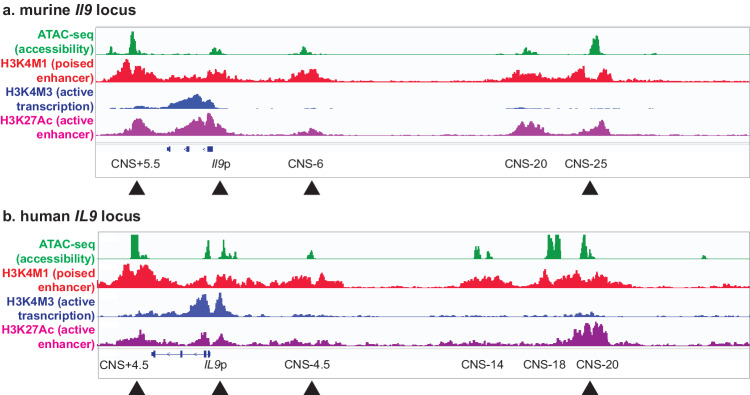
Structure of the murine-extended *Il9* locus and human-extended *IL9* locus with epigenetic marks. **a** Gene tracks show the extended murine *Il9* locus, including the promoter and surrounding putative enhancers. **b** Gene tracks show the extended human *IL9* locus, including the promoter and surrounding putative enhancers. For both human and murine loci, regulatory elements (REs) were identified based on accessibility (ATAC-seq, green), poised enhancer marks (H3K4M1 ChIP-seq, red), active promoter marks (H3K4M3 ChIP-seq, blue), and active enhancer marks (H3K27Ac ChIP-seq, purple). Human and murine orthologs are marked with black triangles. The data were obtained from GSE222910.

The murine *Il9* gene is located on chromosome 13, with the extended *Il9* locus characterized based on CTCF binding sites that mark the boundaries^3,4,7,8^. Within this locus, six putative cis- REs have been identified (Fig. 1a). The best characterized of these is the *Il9* promoter (*Il9*p), also known as conserved noncoding sequence 1 (CNS1). Most Th9-promoting TFs have been shown to target the promoter directly. These include STAT5, STAT6, ETS family TFs (PU.1, ETV5, and ERG), SMAD2, SMAD3, TAK1, OX40, NF-κB, BATF, AP-1, GATA3, IRF1, IRF4, RARα, and DBP^12^. Moreover, many IL-9 enhancers act at least in part by forming 3D interactions, or loops, with the *Il9* promoter^4,7,8^.

Downstream of the promoter, CNS + 5.5 (also called DS or SEa) is positioned ~5.4 kb from the transcriptional start site (TSS) (Fig. 1a)^3,4,7,8^. CNS + 5.5 promotes IL-9 production through OX40 signaling, primarily through RelB/p300-mediated chromatin acetylation. Deletion of CNS + 5.5 significantly reduces IL-9 production in OX40-stimulated murine Th9 cells, while loops between CNS1 (promoter) and CNS + 5.5 form in OX40-induced Th9 cells^4^.

CNS-6 (also referred to as E1 or SEb) is located -6 kb upstream of the *Il9* TSS and binds STAT5, STAT6, and IRF4 to promote IL-9 production (Fig. 1a)^7,13^. Notably, IRF1 also binds to CNS-6, thereby displacing IRF4 to block activating histone modifications and recruit transcriptional repressors^13^. IRF1-mediated repression of IL-9 production occurs downstream of IFN-γ and is STAT1-dependent^13^. CNS-6 deletion reduces IL-9 production in cells differentiated under Th9-promoting conditions in vitro, although the in vivo functions of this cis-RE are not yet known^7^. Further upstream, CNS-20 (also E2) and CNS-25 (also E3 or SEc) are located 20 and 25 kb upstream of the *Il9* TSS, respectively (Fig. 1a). CNS-20 is bound by STAT5 and STAT6, while CNS-25 is bound by many transcription factors, including BATF, IRF4, STAT5, STAT6, GATA3, and Foxo1. Deletion of CNS-20 and CNS-25 reduces Th9 differentiation; CNS-25 is also critical for IL-9 production by mast cells and basophils but does not affect IL-9 production in innate lymphoid cells (ILCs)^3,7,8^. Nonetheless, CNS-25-deficient mice are protected from chronic airway inflammation and anaphylaxis^3,8^. Importantly, looping between CNS-25 and the *Il9* promoter is stronger in Th9 cells than in Th2 cells. Moreover, while CNS-25 is an *Il9* enhancer in Th9 and Th17 cells, it acts as an epigenetic silencer in Th2 cells^8^. The mechanisms underlying this bifunctionality are unclear, but they could involve context-dependent binding of Th9-promoting TFs and Th2-promoting/Th9-repressing TFs.

### The human *IL9* locus

The human *IL9* locus is located on chromosome 5 and has about 55% homology to the murine *Il9* locus^14^. The extended locus contains six putative cis-REs (Fig. 1b). A downstream RE (CNS + 4.5, also known as DS) is located 4.5 kb upstream of the Transcription Start Site (TSS)^2,13^. In addition to the promoter (*IL9*p), there are four upstream elements^2^. CNS-4.5 (also known as E1) is located 4.5 kb upstream of the TSS and is homologous to murine CNS-6, while CNS-14 (also known as E2) is positioned 14 kb from the TSS and shares homology with murine E2^2^. The upstream RE CNS-18 (also known as E3) is homologous to murine CNS-25. Deletion of CNS-18 in human Th9 cells reduced IL-9 expression, while the production of other cytokines was unaffected^8^. This included other type 2 cytokines (IL-4, IL-5, IL-13), although the genes encoding these cytokines are in a cluster approximately 3 mb downstream of the *IL9* locus. Another previously uncharacterized element (CNS-20, also known as E4) is located 20 kb upstream of the TSS (Fig. 1b) and demonstrated Th9 cell-specific accessibility compared to naive cells and other subsets^2^. While Th9-specific chromatin architecture and homology to murine functional enhancers suggest that CNS + 4.5, CNS-4.5, CNS-14, and CNS-20 have critical regulatory roles, functional studies will be needed to address this question more definitively.

## Interactions between IL-9-modulating transcription factors and the extended *IL9* locus

One of the key mechanisms of enhancer-mediated gene regulation involves the recruitment of activating TFs^15^. TF binding to these noncoding DNA sequences results in formation of 3D loops and recruitment of transcriptional machinery. These mechanisms play a pivotal role in the control of lineage-specific gene expression in T cells^15^. A large network of IL-9-inducing TFs binds to the *Il9/IL9* locus to modulate transcription; this includes engagement of key noncoding cis-REs.

### STAT family

STATs are critical signal-dependent TFs that guide T cell development and subset commitment; STAT5 and STAT6 strongly induce Th9 differentiation downstream of IL-2 and IL-4, as well as promoting IL-9 production from other cell types like ILC2s^6,7,10,15–19^. STAT5 is also activated downstream of other Th9-promoting signals, including Itk, although this is also at least partly mediated by IL-2^18^. STAT5 and STAT6 promote not only Th9 differentiation but also IL-9 production in committed Th9 cells, including innate-like bystander activation of recently activated resting human and mouse Th9 cells^2,6^. IL-2/STAT5 signaling also induces IL-9 in ILC2s, while STAT5 promotes IL-9 production downstream of TNF-α in CD8^+^ Tc9 cells^19,20^. Other STAT5-inducing cytokines like IL-7 and IL-15 have not been found to directly induce IL-9, although IL-7 can indirectly induce IL-9 in ILC2s by enhancing the effect of IL-33^21^.

**Fig. 2:**
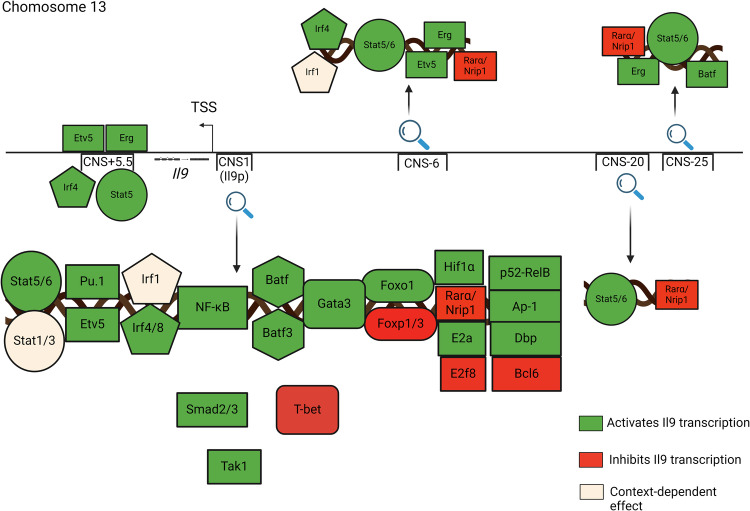
Epigenetic regulation of the extended *Il9* locus by Th9-modulating transcription factors. Schematic shows the 5 putative regulatory elements (REs) that promote *Il9* transcription. Various transcription factors (TFs) that bind to *Il9*-promoting REs are shown, together with their reported RE associations. *Il9*-modulating TFs are reported to promote (green) or repress (red) IL-9 production, or to have context-dependent effects (yellow). Some IL-9-modulating TFs are not reported to directly target the *Il9* locus; these TFs are schematically represented as proximal to, but not associated with, *cis*-REs.

**Fig. 3:**
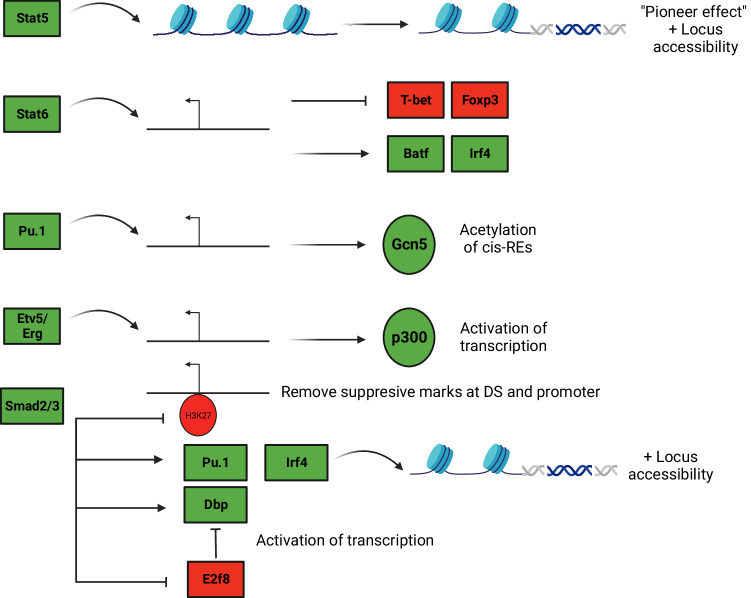
Main mechanisms of action employed by *Il9*-modulating transcription factors. IL-9-regulating transcription factors (TFs) are reported to regulate IL-9 expression via several mechanisms of action. These include direct binding, modulation of accessibility, histone modification, and induction or repression of other Il-9-modulating TFs. In this schematic, the curved arrows indicate that a TF directly targets—or binds to—the *Il9* locus. Activating or inhibitory effects on other proteins are shown by straight arrows or lines, respectively.

STAT5 binds all *IL9* REs, including the promoter and upstream/downstream enhancers (Fig. 2)^6,7^. Consecutively activated STAT5 stimulated >3-fold induction of the *Il9* promoter element, >2-fold induction of CNS-6 and CNS-20, and <1.5-fold induction of CNS-25, establishing that these are STAT5-responsive REs^6,7^. In mast cells, STAT5 binds CNS-25 preferentially to the *Il9* promoter, suggesting that STAT5-enhancer interactions may be different in various cell types^3^. Mechanistically, STAT5 promotes *Il9* locus accessibility and modulates the binding and expression of other transcription factors. STAT5 acts as a “pioneer” TF that is required for other IL-9-inducing TFs like BATF to bind the *Il9* locus (Fig. 3)^6^. STAT5 signaling also controls the balance between Th9 and Th17 differentiation. Blocking STAT5 promotes Th9 plasticity towards a Th17-like phenotype via STAT3-independent and RoRrץt- and BATF-dependent mechanisms^10^. STAT5 binding to the *Il9* promoter also competes with the actions of the IL-9-repressing TF BCL6^22^.

STAT6 is activated downstream of IL-4 signaling and binds at REs throughout the *Il9* locus, like STAT5 (Fig. 2)^6,7^. At the Il9 promoter, STAT6 inhibits the expression and binding of Th9-repressing transcription factors like T-bet and Foxp3 (Fig. 3)^6,12,23^. STAT6 is also required to induce BATF, which promotes Th9 development^6,12,23^. IRF4 directly promotes Th9- and ILC2-derived IL-9 production: STAT6 increases IRF4 expression and function (Fig. 3)^23,24^.

The effects of STAT1 and STAT3 on IL-9 are less straightforward and may be context- or species-dependent. In some murine models, STAT1 induces IRF1, which targets the *Il9* promoter to induce IL-9 (Fig. 2)^25^. Conversely, IL-27/STAT1 signaling represses the differentiation of IL-9-producing T cells, while human *STAT1* gain-of-function mutations repress IL-9 production via induction of T-bet^12,26^. STAT3 interferes with STAT5 activation to suppress murine Th9 differentiation; it is not clear whether STAT3 targets *Il9* REs^16^. By contrast, STAT3 promotes the differentiation of IL-9^+^ human T cells^26^. The reasons for these species-specific effects are unclear but could involve repressing STAT1 to induce IL-9^26^.

### ETS family

The ETS (E26 transformation-specific) family includes three TFs that promote the differentiation of IL-9-producing CD4^+^ T cells: PU.1, ETV5 (ETS translocation variant 5), and ERG (Ets-related gene)^12,27,28^. PU.1 is the first TF found to directly interact with the *Il9* promoter (Fig. 2) and was initially designated as a master TF for the Th9 lineage^29^. However, subsequent studies revealed that Th9 differentiation can proceed in the absence of PU.1, albeit at a much lower efficiency than in PU.1-sufficient cells^27^. PU.1 acts primarily during the early stage of Th9 differentiation; its expression decreases during the later stage of differentiation^12^. PU.1 binds the *Il9* promoter and induces GCN5, a histone acetyltransferase (HAT) that acetylates key lysines on histones H3 and H4, thereby activating critical *cis*-REs (Fig. 2)^30^. PU.1 may also promote Th9 identity by suppressing genes essential for the development of other subsets, including Th2 and Tfh (T follicular helper) cells^12^.

ETV5 is another ETS family TF with a role in the differentiation of IL-9^+^ T cells: deletion of both PU.1 and ETV5 reduces IL-9 production more than individual deletion of each gene^27^. While *Spi1* (encodes PU.1) expression peaks early during Th9 development, *Etv5* expression increases downstream of IRF4 and STAT6 during the later stages of Th9 differentiation^27^. In addition to binding the *Il9* promoter, ETV5 also targets CNS + 5.5 (DS) and CNS-6 (E1) and recruits p300 to activate transcription (Figs. 2 and 3)^27^.

A third member of the ETS family, ETS-related gene (ERG), also uses epigenetic mechanisms to induce IL-9 production and differentiation of IL-9-producing T cells^28^. ERG targets the *Il9* promoter, CNS + 5.5 (DS) and CNS-25 (E3) in naive T cells; recruitment increases at CNS-6 (E1) during Th9 differentiation (Fig. 2). In late stages of differentiation (d5, mouse), ERG recruitment at the *Il9* promoter is significantly higher in Th9 cells than in Th0 or Th2 cells, which do not produce substantial amounts of IL-9. Like ETV5, ERG promotes p300 recruitment during the later stages of Th9 differentiation (Fig. 2)^28^. Conversely, during the early stages of Th9 differentiation, the expression of other ETS-family TFs like PU.1 is upregulated. These TFs can partially compensate for ERG, suggesting that they cooperatively regulate IL-9 production^28^.

### SMADs

Like IL-2 and IL-4, TGF-β is a critical Th9-promoting cytokine^12^. When TGF-β engages its receptor, SMAD2 and SMAD3 are phosphorylated, associate with their common partner SMAD4, and translocate to the nucleus to drive the expression of downstream genes^12^. SMAD2/3 are critical for TGFβ-dependent IL-9 production but are dispensable for other IL-9-inducing cytokines like IL-4 and IL-1β^31^. Downstream of TGF-β, SMAD2 and SMAD3 do not directly bind the *Il9* locus but are still required to displace EZH2 and remove suppressive H3K27 modifications at the *Il9* promoter and CNS + 5.5 (DS) (Fig. 2)^32^. Several indirect mechanisms have been implicated in SMAD2/3-mediated *Il9* epigenetic regulation. Phosphorylation of Serine 213 within the linker region of SMAD3 induces IL-9 by inducing the Albumin D-site-Binding Protein (DBP), a Proline and Acidic amino acid-Rich basic leucine ZIPper (PAR bZIP) TF that directly targets the *Il9* promoter^33^. SMAD2 and SMAD/3 also modulate the binding of PU.1 and IRF4 to the extended *Il9* locus, although they do not directly induce these TFs^32^. Indeed, IRF4 cannot induce *Il9* transcription in the absence of SMAD2/3, suggesting that SMAD2 and SMAD/3 are required for IRF4 to regulate *Il9* locus epigenetics (Fig. 3)^32^.

### TAK1

TGF-β not only phosphorylates Smad2/3 but also triggers the activation of TGF-β activated kinase 1 (TAK1)^34^. Inhibition of TAK1 blocks the development of IL-9-producing CD4^+^ T cells but not the differentiation of other subsets^34^. TAK1 does not regulate PU.1 or IRF4 but rather inhibits the DNA-binding *Il9* transcriptional repressor ID3, thereby promoting IL-9 production^34^. ID3 prevents E2A and GATA3 from binding the *Il9* promoter, although it is unclear whether these mechanisms also regulate other *Il9* cis-REs^34^. TAK1 also inhibits the histone deacetylase SIRT1, which represses the IL-9-inducing mTOR-HIF1α pathway^35^. Like E2A and GATA3, HIF1α directly targets the *Il9* promoter (Fig. 2); its role at other CNS regions could be an area of future investigation^35^.

### NF-κB

Multiple NF-κB-activating signals promote Th9 differentiation and induce IL-9 in other cell types^12^. For example, lipopolysaccharide enhances IL-9 production in activated mast cells^36^. In T cells, NF-κB (p65) binds the *Il9* promoter to induce transcription downstream of T cell receptor (TCR) activation (Fig. 2)^37^. IL-9-promoting cytokines that signal through NF-κB include IL-1β, IL-33, and IL-36; IL-2 is also reported to promote IL-9 production via NF-κB induction^31,38–42^. In addition to directly targeting the *IL9* locus, IL-1β promotes IL-9 production by suppressing the expression of the IL-9-inhibitory TF BCL6^39,43^.

The TNFR superfamily contains many IL-9-inducing factors that act through NF-κB^20,44–47^. TNF-α induces IL-9 in CD4^+^ T cells, CD8^+^ T cells, and eosinophils^20,48^. OX40 is a costimulatory molecule expressed by activated CD4+ and CD8 + T cells; its ligand (OX40L) is exclusively expressed by antigen-presenting cells (APCs). Downstream of OX40L-OX40, p52-RelB directly binds the *Il9* promoter, inducing IL-9 independent of the PU.1 and STAT TFs (Fig. 2)^44^. Although OX40 also promotes canonical (p50-RelA) NF-κB activation, this pathway is dispensable for IL-9 production^44^. OX40-OX40L signaling also induces Batf3, another IL-9-promoting TF (Fig. 2)^49,50^. Other IL-9-inducing TNFR family members include Fas, TL1A, and GITR (glucocorticoid-induced TNF receptor-related)^20,44–47^. Like OX40, GITR is a costimulatory molecule that induces IL-9 through TRAF6-NF-κB^47^. TL1A acts via STAT5 and BATF family TFs; although TL1A also activates NF-κB, this pathway may be less critical for TL1A-induced IL-9 production^46^. The TNFR family member Fas activates PKC-β, which both activates NF-κB and inactivates NFAT1. This has the dual effect of enhancing IL-9 production and creating a negative feedback loop that constrains Fas-induced Th9 differentiation^45^.

### BATF family

The BATF family is composed of three members: BATF, BATF2, and BATF3, all expressed primarily in T and B cells^6,49,51^. IL-9-promoting inducers of BATF and BATF3 include TCR, OX40, TL1A, and IL-4/STAT6^6,49–51^. BATF and BATF3 are highly homologous and can compensate for each other to induce IL-9 through similar epigenetic mechanisms^49^. BATF acts by binding the *Il9* promoter and CNS-25 but cannot increase the accessibility of *Il9* chromatin (Fig. 2)^6^. Consequently, BATF can only activate transcription when the locus is already poised by other TFs like STAT5^6^. BATF3 also targets the *Il9* promoter, where it interacts with IRF4 to form a complex that collaboratively enhances transcription (Fig. 2)^49^. Notably, PU.1 binding remains unaltered in *Batf-*deficient mice, indicating that PU.1 and BATF function independently^50^.

### FOX family

Foxo1, a member of the forkhead box O (Foxo) family, plays a pivotal role in diverse cellular processes like cell survival, apoptosis, and T cell development^52^. During T cell differentiation, Foxo1 is phosphorylated and inactivated by PI(3)K/AKT signaling; IL-7-driven dephosphorylation activates Foxo1, as does TGF-β/Smad3 signaling^52,53^. After activation, Foxo1 binds and transactivates the *Il9* and *Irf4* promoters (Fig. 2), a process synergistically enhanced by IRF4-Foxo1 binding in a feed-forward loop^52^. STAT5 can also interact with Foxo1 signaling to induce IL-9 production by promoting permissive epigenetic changes like p300 recruitment and histone acetylation at the *Il9* locus^54^. Simultaneously, Foxo1 inhibits the Th17 cell program by targeting Rorγt to modulate Th9-Th17 plasticity. Other FOX family TFs with a role in IL-9 regulation include Foxp1, which binds and negatively regulates the *Il9* promoter, competing with Foxo1^54^. Foxp3 can also suppress IL-9 via incompletely understood mechanisms involving GITR and STAT6 (Figs. 2 and 3)^12^.

### IRFs

Interferon-regulatory factor 4 (IRF4) induces IL-9 in multiple cell types downstream of diverse stimuli including IL-33, thymic stromal lymphopoietin (TSLP), TGF-β, TL1A, and IL-4^12,24,55^. IRF4 directly targets the *Il9* promoter and CNS + 5.5, increasing locus accessibility (Fig. 2)^13,50^. Downstream of Smad3, IRF8 also targets the *Il9* promoter and forms complexes with other TFs like BATF and PU.1 (Fig. 2)^56^. These complexes induce IL-9 production in Th9 cells and may have a role in other cell types^56^. IRF1 directly induces IL-9 downstream of IL-1β but represses IL-9 downstream of IFN-γː in both cases, IRF1 modulates accessibility of the *Il9* locus^13,25^. These disparate results may be related to interactions with other TFs like NF-κB.

### GATA3

In addition to activating STAT6, IL-4 induces GATA3, which promotes IL-9 production in T cells and potentially in other cell types^23^. Other TFs like the DNA-binding inhibitor Id3 modulate IL-9 indirectly through GATA3. Like many other TFs, GATA3 targets the *Il9* promoter, although its binding has not been investigated at other cis-REs (Fig. 2)^34^.

### PPAR-γ

Peroxisome proliferator-activated receptor γ (PPAR-γ) is a lipid-activated TF with a well-established role in Th2 cells^57,58^. Because of the overlap in Th2/Th9 identity and function, several studies have subsequently investigated the role of PPAR-γ in Th2-Th9 plasticity. In allergic subjects, IL-9 production can define a subpopulation characterized by an activation-induced signature and high PPAR-γ expression^58^. In this subpopulation, IL-9 production is transient, with IL-9^+^ cells developing into conventional Th2 cells. Notably, PPAR-γ agonists do not modulate IL-9 or Th2 cytokine production, implicating a ligand-independent mechanism^58^. The mTORC1 signaling pathway may modulate the effects of PPAR-γ on IL-9 production^57^.

### HIF1α

Metabolic regulation is essential for T cell activation and differentiation due to complex and shifting energy requirements, mainly through aerobic glycolysis and oxidative phosphorylation^35,57,59^. HIF1α, a well-known TF, plays a pivotal role in orchestrating metabolic pathways in T cells, transitioning from aerobic to anaerobic metabolism, reducing ATP depletion, preventing the apoptosis of inflammatory effector cells, and modulating inflammatory capacity^60^. In CD4^+^ and CD8^+^ T cells, extracellular ATP promotes IL-9 production through nitric oxide (NO), mTOR, and HIF-1α^61,62^. HIF-1α directly targets the *Il9* promoter (Fig. 2) and regulates *Nos2* to induce NO in a feed-forward loop^60^. HIF-1α can also be activated by EGFR (epidermal growth factor receptor), and the EGFR ligand amphiregulin strongly induces Th9 differentiation downstream of IL-33 and TSLP^60^.

### Vitamin receptors (RAR and VDR)

Vitamins A and D metabolites signal through nuclear receptors that act as TFs to modulate gene expression^7^. The vitamin D metabolite calcitriol engages VDR (vitamin D receptor), which interacts with PU.1 to block its binding to the *Il9* promoter and repress the differentiation of IL-9-producing T cells (Fig. 2)^63^. Vitamin A metabolism produces several immunomodulatory metabolites, including retinoic acid (RA), which represses Th9 differentiation through RA receptor alpha (RARα)^7^. RARα recruits the corepressor NRIP1 to the *Il9* promoter and upstream enhancers, reducing chromatin accessibility. Concurrently exposing Th9 cells to both metabolites negates this epigenetic repression by altering VDR-RXR (retinoic X receptor) interactions and HDAC recruitment^64^.

### Other repressive TFs: TBX21, BCL6, and E2F8

TBX21, or T-bet, suppresses Th2 and Th9 differentiation while promoting Th1 differentiation; T-bet also suppresses IL-9 in ILCs^26,65^. While T-bet binds to key enhancers in different T helper subsets, its actions at the *Il9* locus are unknown^66^. BCL6 is an IL-9-repressing TF that binds the *Il9* promoter near the STAT5/6 binding site (Fig. 2)^22,39,43^. IL-2, IL-1β, and IL-21 modulate IL-9 expression by altering the relative expression of BCL6 and STAT5^22,39,43^. Similarly, the IL-9-repressive TF E2F8 competes with DBP at the *Il9* promoter: Smad3 represses E2F8 while inducing DBP (Fig. 2)^33^.

Together, these studies demonstrate that modulation of cis-REs within the *Il9* locus underlies critical regulatory mechanisms involving a broad cross-section of TFs and stimuli.

## Clinical/epidemiological associations of the extended *IL9* locus with human disease

### Allergy and atopic dermatitis

An extensive body of literature links IL-9 to type 2 (allergic) inflammation (Table 1). Expression levels of IL-9 and its receptor are increased in both murine allergy/asthma models and human subjects with allergy^1^. Blockade of IL-9 and its receptor improve pathology in murine disease models, whereas transfer of IL-9-producing cells exacerbates pathology^1^. While IL-9 blockade was not successful in early asthma clinical trials, many of these trials were performed in the pre-endotyping era, leading some investigators to propose newer trials in “IL-9^high^/Th9^high^” subjects^2^. Various single nucleotide polymorphisms (SNPs) in *IL9* and the gene encoding its receptor, *IL9R*, are linked to allergic disease risk – including SNPs within putative IL9 cis-REs, although linkage disequilibrium can make it difficult to identify disease-causal SNPs within an extended locus^67^. For example, one disease-associated polymorphism, rs11741137, is located within CNS + 4.5 (DS)^68^. In subjects with proven housedust mite allergy, the T allele is associated with an increased risk of housedust mite induced severe asthma exacerbation^68^. Similar results were observed for patients with the A allele of rs2069885, located within the *IL9* promoter. Also within the IL-9 promoter, the G allele/GG genotype at rs1859430 is associated with increased asthma risk, whereas the C allele/CC genotype at rs2066758 reduces disease risk^69^. The *IL9* promoter SNP rs2069885 and intronic SNP rs2069882 are both associated with sex-specific differences in asthma risk^70^.

**Table 1:** Disease-associated SNPs within the *IL9* locus, together with mapped location (hg38).

SNP	Association	Location
rs2069885	asthma, TNFi response, macular degeneration, laryngeal SCC, sex-specific RSV severity, sex-specific lung function	promoter
rs1859430	asthma, macular degeneration, laryngeal SCC	promoter
rs2066758	Asthma	promoter
rs31563	atopic dermatitis, coronary artery disease, cholesterol levels	promoter
rs1799962	sex-specific differences in RSV bronchiolitis	promoter
rs2069868	coronary artery disease	promoter
rs2069870	cholesterol levels, laryngeal SCC	promoter
rs2069884	macular degeneration, laryngeal SCC	Intronic noncoding region
rs31564	coronary artery disease	Intronic noncoding region
rs55692658	coronary artery disease	Intronic noncoding region
rs2069882	sex-specific differences in asthma	Intronic noncoding region
rs11741137	asthma exacerbation, macular degeneration, laryngeal SCC	CNS-4.5
rs1859428	malignant melanoma, myocardial infarction	CNS-20
rs740002	cutaneous malignant melanoma	CNS-14
rs3093467	atopic dermatitis (in association with rs31563)	IL9R (not IL9)

Table depicts single nucleotide polymorphisms (SNPs) within the human *IL9* locus that are associated with human diseases, as well as the location of each SNP and the associated disease.

*TNFi* tumor necrosis factor inhibitor, *SCC* squamous cell carcinoma, *RSV* respiratory syncytial virus, *CNS* conserved nucleotide sequence, *IL9R* IL9 receptor

While *IL9* locus genetics have been most extensively studied in asthma, increased IL-9 expression is also seen in atopic dermatitis (AD, Table 1)^1^. The rs31563 SNP located within the *IL9* promoter increases allergic-type AD susceptibility, whereas the rs3093467 *IL9R* SNP is associated with non-allergic dermatitis^71^. Gene-gene interaction analysis suggests these two SNPs synergistically promote AD by combining the rs31563 GG and rs3093467 TT genotypes^71^.

### Autoimmunity

In addition to allergic disease, increased IL-9 expression, Th9 cell expansion, and IL-9^+^ ILC2 expansion have also been reported in autoimmune diseases like inflammatory bowel disease (IBD), systemic sclerosis, rheumatoid arthritis (RA), psoriatic disease, and vasculitis^72–76^. IL-9 promotes antibody production and B cell proliferation, suggesting it may have a pathogenic role in autoimmune diseases^75^. In IBD, Th9 cells are elevated within the intestinal mucosa and prevent wound healing by promoting intestinal cell permeability^74^. In psoriatic disease, IL-9^+^ cells infiltrate skin and joints, and IL-9 induces pathogenic IL-17 expression^77,78^. The role of IL-9 in RA and scleroderma is less clear: IL-9 may promote wound healing and reduce fibrosis in some contexts, but IL-9 is also reported to induce fibrosis and worsen joint inflammation^72,75,76,79–81^. In multiple sclerosis, IL-9 is elevated within the central nervous system and reduces macrophage activation^82^.

Because ongoing studies are still defining the role of IL-9 in autoimmune diseases, the role of *IL9* epigenetics in autoimmunity is not as well investigated as it is in allergy (Table 1). In patients with axial spondyloarthropathy, the *IL9* promoter SNP rs2069885 A allele is associated with a favorable response to tumor necrosis factor (TNF) inhibitors^83^. Epigenetic modulators of IL-9 like PU.1 are implicated in murine models of autoimmunity including IBD and RA^74,80^. The rs3093457 *IL9R* SNP is also associated with RA, and further studies are needed to probe whether interactions with *IL9* SNPs promote RA, as seen for *IL9R* SNPs in allergy^84^.

### Cancer

IL-9 has both tumorigenic and antitumorigenic properties depending on the type of cancer and the tumor microenvironment. As a lymphocyte growth factor, IL-9 promotes tumorigenesis in many hematological malignancies^85^. These include T-cell malignancies like anaplastic large-cell lymphoma and human T-cell leukemia virus type 1 (HTVL-1) transformation, as well as B cell malignancies like Hodgkin’s lymphoma^85^. Yet, in the setting of hematopoietic stem cell transplantation, IL-9 can promote graft vs. leukemia-mediated antitumor effects^86^. IL-9 also has profound antitumor activity against solid tumors^87^. This is at least partly through stimulation of cytotoxic CD8+ lymphocytes, an effect that has been leveraged to engineer T cells bearing synthetic orthogonal IL-9 receptors with enhanced antitumor activity^88^.

Supporting the critical role of IL-9 in antitumor immunity, five SNPs within the *IL9* promoter and CNS + 4.5 have been linked to laryngeal squamous cell carcinoma (LSCC, Table 1)^89^. The A-G-C-G-G haplotype of rs1859430-rs2069870-rs11741137-rs2069885-rs2069884 reduces disease risk, while the AA genotype at rs1859430 is associated with poor survival^89^. The G allele and GG genotype of rs1859430 in the *IL9* promoter also increase the risk of recurrent pituitary adenoma (PA)^90^. Upstream of the *IL9* promoter, rs740002 and rs1859428 within CNS-14 and CNS-20 (E2-E3) are associated with malignant melanoma, particularly in patients with pro-oncogenic *CDKN2A* mutations^91^. Together, these studies suggest that noncoding *cis*-REs modulate IL-9 expression, thereby affecting homeostatic tumor surveillance.

### Other diseases

In addition to modulating immune cell signaling and function, IL-9 can also affect non-hematopoietic cells like keratinocytes, smooth muscle cells, and glial cells – although these roles are not well characterized^1^. These effects may underlie the associations of IL-9 with non-immunologic diseases; alternatively, IL-9 may promote subclinical immune dysfunction in patients with these diseases^1^. Age-related macular degeneration is negatively associated with haplotypes A-G-C-G-G and G-A-T-A-T in rs1859430, rs2069870, rs11741137, rs2069885, and rs2069884 (Table 1)^92^. Three of these SNPs are in the *IL9* promoter, one is in CNS + 4.5, and one is in an intronic noncoding region. Cholesterol levels are associated with two *IL9* promoter SNPs, while coronary artery disease is associated with SNPs in the IL9 promoter and intronic noncoding regions^93,94^. This is consistent with the observation that IL-9 worsens murine atherogenesis, while serum IL-9 is elevated in patients with atherosclerosis^95^.

## Conclusions

The *IL9* locus is a complex regulatory region comprising multiple cis-REs that interact with each other to modulate IL-9 production. Although most studies have been done in murine cells, the human *IL9* locus contains putative REs homologous to major murine *Il9* promoter and enhancer regions. Nonetheless, the human *IL9* locus has some unique REs and structural features, so further studies should focus on characterizing IL-9 epigenetic regulation in human cells. Although several cell types can produce IL-9, most epigenetic studies have been done in Th9 cells. In Th9 cells, the *IL9* locus exhibits a unique dynamic accessibility that permits innate-like IL-9 production in recently activated Th9 cells but also prevents sustained nonspecific IL-9 production over time. A complex network of TFs bind to and regulate the *IL9* locus, including ETS family TFs, STATs, IRFs, NF-kB, SMADs, FOX family TF, and IRFs^1^. These TFs almost universally bind the *IL9* promoter; some also engage upstream and downstream enhancers, suggesting that they may be involved in 3D chromatin looping.

Epigenetic regulation of IL-9 is seen in murine models of allergic asthma, autoimmunity, antihelminth defense, and antitumor immunity. SNPs within *IL9 cis*-REs are associated with a broad spectrum of inflammatory diseases, as well as nonimmunologic conditions. Together, these studies implicate epigenetic modulation as a core regulatory mechanism governing IL-9 production in the setting of human disease. However, many questions remain unanswered: these include the possibility of cell-specific REs, RE-specific TF binding, and the role of REs found exclusively in the human *IL9* locus. Further investigations of these unanswered questions will provide a more complete view of epigenetic mechanisms governing IL-9 production and a broader understanding of noncoding REs in human physiology, homeostasis, and disease.
